# Integration of Conversion Factors for the Development of an Inclusive eHealth Tool With Caregivers of Functionally Dependent Older Persons: Social Justice Design

**DOI:** 10.2196/18120

**Published:** 2020-08-26

**Authors:** Karine Latulippe, Christine Hamel, Dominique Giroux

**Affiliations:** 1 Laval University Québec, QC Canada; 2 Centre de recherche en santé durable VITAM Quebec, QC Canada; 3 Centre d'Excellence du Vieillissement de Québec Chu de Québec Quebec, QC Canada

**Keywords:** caregivers, aged, help-seeking behavior, community-based participatory research, eHealth, telemedicine, mobile phone

## Abstract

**Background:**

eHealth can help reduce social health inequalities (SHIs); at the same time, it also has the potential to increase them. Several conversion factors can be integrated into the development of an eHealth tool to make it inclusive: (1) providing physical, technical, and financial access to eHealth; (2) enabling the integration of people at risk of SHIs into the research and development of digital projects targeting such populations (co-design or participatory research); (3) promoting consistency between the digital health literacy level of future users (FUs) and the eHealth tool; (4) developing an eHealth tool that is consistent with the technological skills of FUs; (5) ensuring that the eHealth tool is consistent with the help-seeking process of FUs; (6) respecting the learning capacities of FUs; and (7) being sensitive to FUs’ cultural context. However, only little empirical evidence pointing out how these conversion factors can be integrated into an effective eHealth tool is available.

**Objective:**

On the basis of Amartya Sen’s theoretical framework of social justice, the objective of this study was to explore how these 7 conversion factors can be integrated into an eHealth tool for caregivers of functionally dependent older persons.

**Methods:**

This study was based on a social justice design and participant observation as part of a large-scale research project funded by the Ministère de la Famille through the Quebec Ami des Aînés Program. Data were collected by recording the preparation sessions, the co-design and advisory committee sessions, as well as the debriefing sessions. The results were analyzed using Miles and Huberman’s method.

**Results:**

A total of 78 co-designers participated in 11 co-design sessions, 24 preparation sessions, and 11 debriefing sessions. Of the 7 conversion factors, 5 could be explored in this experiment. The integration of conversion factors has been uneven. The participation of FUs in the development of the tool supports other conversion factors. Respecting the eHealth literacy level of FUs means that their learning abilities and technological skills are also respected because they are closely related to one another and are therefore practically difficult to be distinguished.

**Conclusions:**

Conversion factors can be integrated into the development of eHealth tools that are intended to be inclusive and contribute to curbing SHIs by integrating FU participation into the tool design process.

## Introduction

### Background

Do you have access to your digital health record? What mobile apps do you use? eHealth, or any other digital tool used to take care of our health, is an integral part of our lives. However, a segment of the population cannot use these means to take care of their health, which leads to social health inequalities (SHIs). SHIs represent, for groups of people, the difference in the prevalence of disease and mortality rates due to unfair and modifiable social factors [1]. eHealth can exacerbate SHIs due to the digital divide [2]. The term *digital divide* evokes the separation between those who have access to technologies, such as computers, mobile phones, or the internet, and those with no such access, especially low-income individuals [3-5]. This concept also highlights the knowledge gap between users. Furthermore, this term refers to the notion of significant (or universal) access, which includes equipment, internet connection, skills development, technical assistance, and appropriate content, meaning health information that is comprehensible and useful for disadvantaged populations. The concept of digital divide also includes geographical location, behavior for searching information, confidence about private life and institutional policies, language, incapacity, and the lack of cultural sensitivity [3,5-7]. People who are in poor health condition and hence at higher risk of SHIs are also more likely to experience this digital divide [2]. eHealth makes a genuinely positive contribution to reducing SHIs by providing effective access to health services [8] anytime and anywhere while reducing stigma [9], which has led to a health justice issue.

### Conceptual Framework

**Figure 1:**
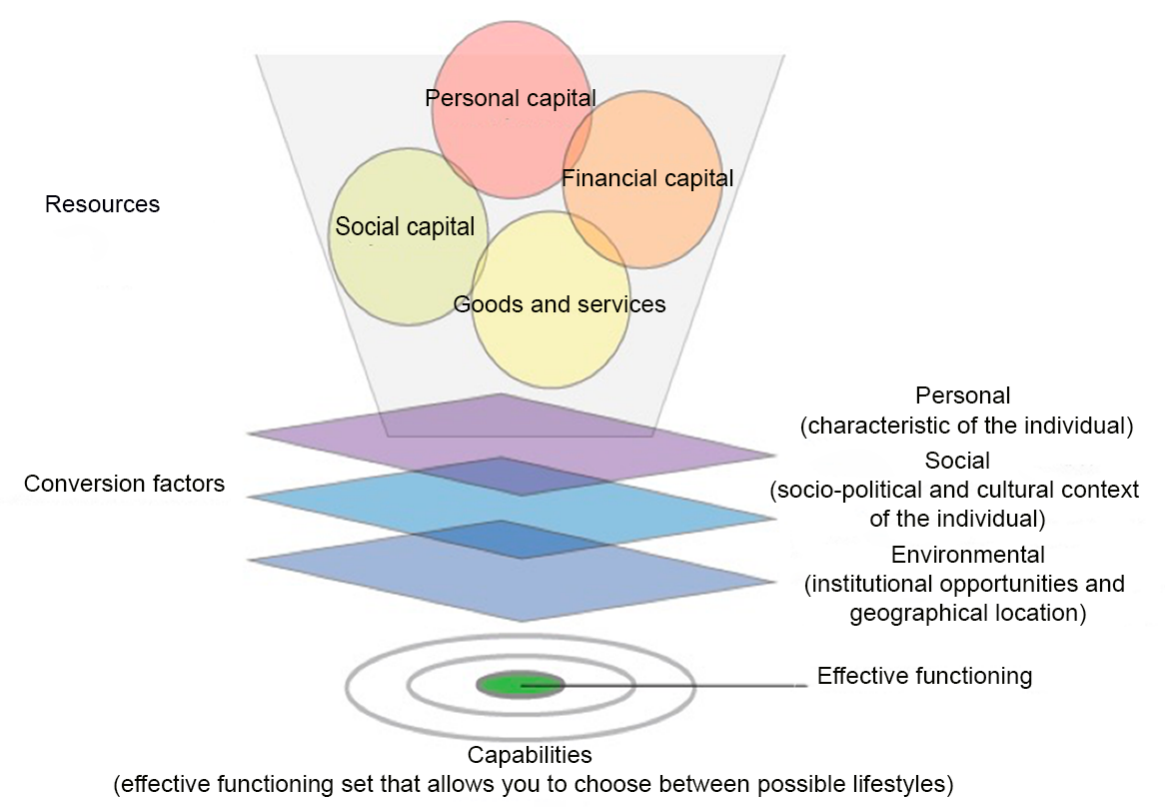
Representation of the Capability Approach.

The capability approach proposed by Amartya Sen provides an interesting theoretical framework for addressing SHIs in eHealth [10]. His approach is different from the more classical school of thought regarding the notion of equality (utilitarianism vs egalitarianism), understood as an individual’s freedom to choose a course of life that he or she has good reasons to value (ie, their capabilities) [11]. It is, therefore, an opportunity for individuals to perform an activity that makes sense to them. They must be able to convert their resources and formal rights into effective functioning. Subsequently, they can choose whether or not to engage in activities that are conducive to achieving the lifestyle that they have chosen (capability). The capability approach is illustrated in Figure 1.

Resources that can be mobilized refer to personal, social, and financial capital as well as goods and services [11]. However, even if all individuals had the same resources, human diversity, recognized by Sen as being ubiquitous, means that the mobilization of these resources would vary from one individual to another and would not necessarily lead to effective functioning [10]. Conversion factors are the different personal, social, and environmental characteristics of a person that positively or negatively affect their ability to convert their resources and formal rights into effective functioning [12]. Differences in conversion factors lead to different (or unequal) degrees of freedom in achieving capabilities [12]. In other words, conversion factors can be viewed as intervening variables or categories of intervening variables that may support or hinder effective functioning. Effective functioning represents the accomplishments or achievements of an individual [13].

There is a significant consistency between the concepts of SHIs and the capability approach. SHIs can result in a limited ability to take care of one’s health. Although Sen refuses to establish a list of capabilities, this was done by Nussbaum [14], who identified a list of 10 basic human abilities, one of which, labeled *life*, refers to being able to live a *normal* life and avoiding premature death [15]. This is fully in line with the idea of combating SHIs. In individuals at risk of SHIs, one or more characteristics are associated with variations in resources such as low income, living alone or in a single-parent situation, precarious occupational status, belonging to an ethnic minority, and poor health literacy or education level [2]. Health services and eHealth can also fall within the resources category that Sen refers to. Although access to health services must be free and universal (formal rights), many negative conversion factors can hinder the mobilization of resources, already marked by vulnerabilities. The digital divide potentially associated with the use of eHealth is an example of a negative conversion factor that can be broken down into more detailed ones, namely, difficulty in initiating and completing the process of help-seeking, difficulty in accessing eHealth, limited ability to use technology, limited ability to fully understand what is said and written about health, and learning difficulties [2,16]. Promising strategies for the development of an eHealth tool that takes into consideration SHIs are potentially positive conversion factors.

### Conversion Factors

On the basis of this conceptual framework of social justice [10], the following 7 conversion factors conducive to curbing SHIs in eHealth projects have been identified: (1) providing physical, technical, and financial access to eHealth; (2) enabling the involvement of people at risk of SHIs in the research and development of digital projects that concern them (co-design or participatory research); (3) promoting consistency between the level of digital health literacy of future users (FUs) and the eHealth tool; (4) developing an eHealth tool that is consistent with the technological skills of FUs; (5) ensuring that the eHealth tool is consistent with the help-seeking process of FUs; (6) respecting the learning capacities of FUs; and (7) being sensitive to FUs’ cultural context [2].

#### Providing Physical, Technical, and Financial Access to eHealth

In Quebec, in 2018, 95% of adults had at least one electronic device (computer, smartphone, tablet, connected exercise bracelet, or smartwatch) [17], and 92% of them had internet access at home [17]. In Canada, almost all Canadians aged under 45 years use the internet daily [18]. This decreases to 35% as Canadians advance in age, that is, 75 years and above [18]. In addition, education and income are important indicators of internet use among older persons. Globally, North America and Europe are the continents where the internet penetration rate exceeds 85%, followed by Latin America, Australia, and the Middle East with rates ranging from 65% to 70% [19]. Asia and Africa have a penetration rate of 54% and 40%, respectively [19]. Although inequalities are present around the world, they seem to be less with regard to access in Quebec. However, these data should be used with caution. In a 2018 research project on the use of the tablet computer to prepare for hospital discharge, one patient was unable to participate in the research project because the internet and a cellular network were not available in her municipality (paper in preparation). A Quebec project called *Régions branchées* aims to provide complete internet access in Quebec [20]. However, this objective appears ambitious considering the vastness of the province. In addition, the costs associated with internet connection or technical assistance, which may sometimes be necessary, can force families and people experiencing poverty to make difficult choices in their budget management. More and more public establishments (shopping centers, hospitals, libraries, etc) offer free internet access. However, this can lead to confidentiality issues, especially when the search subject is related to health [21]. A Canadian program called Connected Families attempts to address this problem by providing Canadian families living in poverty with access to high-speed internet packages at a cost of Can $10 (US $7.47) per month [22]. It is not known at this time whether low-income families are using this program.

#### Enabling the Involvement of People at Risk of SHIs in the Development of Digital Projects That Concern Them (Co-Design or Participatory Research)

Involving FUs and a diversity of perspectives, circumstances, capacities, and experiences in the design process increases the likelihood that the tool will meet their needs and preferences [23]. Similar to many participatory methodologies, such as participatory action research, patient-partner approach, community-based research, or co-design, the objective of this study was to involve the people targeted by the research project in the process as early as possible in the hope of obtaining better results for them, including people at risk of SHIs. From Sen’s perspective, any way of looking at a problem (and its solutions) is a social construction that implies the need to include the people concerned [13].

#### Promoting Consistency Between the Level of Digital Health Literacy of FUs and the eHealth Tool

eHealth literacy was defined as “the ability to seek, find, understand, and appraise health information from electronic sources and apply the knowledge gained to addressing or solving a health problem” [24]. It is very important to present information that can be understood by patients and users of eHealth tools to help them make decisions about their health and benefit from remote intervention programs [25]. People with poor literacy skills are less likely to use health information technologies and have a poorer overall health status and an increased risk of death [26].

#### Developing an eHealth Tool That Values Technological Skills of FUs

Technological skills or abilities refer to the use of various software, digital platforms, and apps in educational, professional, or everyday life activities [27]. This may also include activities such as securing personal data and appropriating new technologies [27]. In Quebec, 19% of adults believe that they have poor skills, or they do not use the internet [28]. Age also seems to affect the sense of competence [29]. However, in recent years, internet use doubled from 32% to 68% among Canadians aged 65 years and above [29]. Bowen et al [30] have argued that low technological skills are as important a reason as the cost for not adopting the internet.

#### Ensuring That the eHealth Tool is Consistent With the Help-Seeking Process of FUs

The number of people who look for web-based information about their health problems and available services has increased; however, the need to interact with health professionals remains important [31]. In their study, Lin et al [31] argued that people seek information that was put out, among others, by people who are in the same situation as them; in other words, perceived similarity appears to be more influential than perceived expertise. It is important for FUs to identify the eHealth tools that not only can help them take care of their health but also guide them through their process of seeking formal help.

#### Respecting the Learning Capacities of FUs

Many eHealth tools are intended to offer some form of health education. However, studies show that some of them are less effective because they were not designed on the basis of learning theories [32]. eHealth tools would benefit from including key principles related to effective learning environments to allow FUs to get the most out of the tool to improve their health. These include, among others, fostering positive emotions and motivation by ensuring that FUs feel able to achieve what is expected of them, that they are able to perceive a stable link between their actions and results, that they have a clear vision of the objective, that they feel positive emotions toward the learning activities, and that they give relevance to the task [33]. In addition, it seems important to aim for easy knowledge acquisition by focusing on understanding topics rather than memorization, thereby allowing learners to understand when, where, and why to use information. It also seems important to enhance the adaptive skills of the learners, that is, the ability to creatively use the topic mastered in contrast to simply applying the subject matter effectively by supporting metacognition and a reflective view of learning [34]. More specifically, with regard to the use of digital technology, active cognitive processing must be supported without overloading the learner’s cognitive abilities with computer technology [35].

#### Being Sensitive to the FUs’ Cultural Context

People may not feel attracted to the eHealth tool if it does not match their beliefs, values, and habits. The use of photographs representing FUs and a variety of testimonies can support the cultural aspect of the tool [36,37].

### Objectives

On the basis of Amartya Sen’s theoretical framework of social justice, the objective of this paper was to explore how conversion factors can be integrated into an eHealth tool through a co-design project for caregivers of functionally dependent older persons.

## Methods

### Study Design

To attain this goal, the exploration of conversion factors will be carried out through a field project titled “Better meeting the needs of caregivers in providing safe home care for the functionally impaired older persons,” which the research team informally refers to as *the QADA project* in recognition of the fact that it is funded by the Ministry of Families as part of the Age-Friendly Quebec Program (QADA). The project is led by a group of researchers whose intention is to include the social justice perspective in their project (see the protocol of this project for more details) [38]. The purpose of the QADA project is to develop an eHealth tool that facilitates the process of help-seeking for caregivers of functionally dependent older persons. The QADA project is based on a participatory design, more specifically, a co-design approach, and therefore meets the conversion factor that involves the participation of FUs in the development of the eHealth tool.

This study is qualitative in nature, with what can be described as a social justice design as the concept of social justice, based on the capabilities approach, is involved in all phases of the study [39]. It, therefore, aims to determine ways to integrate conversion factors in the development of the eHealth tool to make it inclusive for all caregivers of functionally impaired older persons.

### Epistemological Posture

The epistemology of this study concurs with that of Miles and Huberman [40] in recognizing that social phenomena exist in a real world where regularities are observed and connections between them are established. Some of these observations, however, are based on human subjective experience. It also relates to the desire for social justice and the restoration of power among individuals. Finally, it supports the idea that knowledge develops in action, and as the purpose of this study was to obtain a solution to the problem, any potentially useful methods have their rightful place in it [41]. Thus, it could be said that this study is rooted in the pragmatic paradigm [42].

### Research Sites

This study was conducted in 11 Quebec regions (Côte-Nord, Mauricie, Centre-du-Québec, Capitale-Nationale, Chaudière-Appalaches, Montérégie, Bas St-Laurent, Gaspésie, Outaouais, Montréal, and Laval). The locations of co-design sessions vary, depending on their availability (eg, municipal or community). The work sessions of the research team were held at the research center, sometimes in person and sometimes via Skype.

### Population, Participants, and Selection Criteria

In this study, all QADA project co-designers were participants and were divided into 4 categories: caregivers, community workers, health and social service professionals (HSSPs), and research team members.

#### Caregivers

The population of caregivers of functionally impaired older persons is particularly interesting as it is a very diversified group of people (ie, rich, poor, young, not so young, having a variety of skills, etc) having in common the role of providing care to another person. In this study, caregivers of a functionally impaired older person are those who provide regular, unpaid assistance to a person aged 65 years and above and are a population group at risk of SHIs. Owing to the nature of their tasks, caregivers are more likely to develop physical and mental health problems [43-45]. Some of them are already at risk for SHIs (eg, low income, mental health issues, immigrant status, etc). Bucki [43] argued that caregivers with the lowest incomes had poorer health (ie, psychological and physical functioning, self-efficacy, lifestyle, family support, social capital, and physical and financial security). In addition, lack of resources, limited access to information, social exclusion, and exposure to harmful environments also affect both the caregivers and the elderly person they support and represent factors that create important SHIs [46]. Factors that increase the risk of burnout among caregivers (ie, ethnicity, language, gender, socioeconomic status, health literacy, age, poor education, history of depression, and high time consumption for care) are virtually the same factors that increase the risk of experiencing SHIs [47-49]. This means that caregivers at risk of burnout who need help are also those who are likely to experience SHIs and to be excluded from eHealth interventions. This has a double impact on social justice by dint of the caregivers’ limited ability to take care of their own health, on the one hand, and to take care of the sick person, on the other hand. The latter is, therefore, also in a situation of injustice. Bucki [15] highlighted, in her study, the importance of continuing the fight against SHIs for caregivers.

#### Community Workers and HSSPs

Given their proximity to caregivers, the possibility of obtaining an additional perspective, and the desire that the tool developed be complementary to what already exists, the choice to integrate community workers and health and social services professionals as co-designers was relevant to the QADA project. Their participation in this study allows us to understand how professionals perceive conversion factors and wish to integrate them into the co-design process.

#### Research Team

The members of the research team are participants, and this is of key importance in this study insofar as the integration of conversion factors must rest on an epistemological and methodological choice made by researchers or designers that must be applied in a realistic and concrete way. Their point of view, which will be largely experiential within the QADA project, is therefore crucial for the implementation of the recommendations resulting from this study.

#### Number of Participants and Selection Criteria

A total of 78 co-designers participated in this project and are detailed as follows:

1. *Caregivers:* 30 caregivers participated in this project. In the context of this project, any person providing unpaid assistance on a sustained (weekly) basis to a functionally impaired older person was considered a caregiver.
2. *Community workers*: 26 community workers participated in this project. They had to provide services or interact directly with caregivers of functionally impaired older persons.
3. *HSSPs*: 18 HSSPs participated in this project. Similar to the community workers, the HSSPs had to provide services or interact directly with the caregivers of functionally impaired older persons. These professionals included nurses, nursing assistants, client care attendants, home care workers, occupational therapists, physiotherapists, physicians, social workers, and psychologists.
4. *Research team members*: The research team of the QADA project initially consisted of 8 coresearchers, whose participation varied based on their availability and their expertise. The members of the research team were involved in all phases of the project, and they included the QADA project director, an anthropologist and professional researcher, a user experience designer, and the author of this paper—a doctoral candidate in educational technology.

In line with the epistemological view of the author of this paper, she was involved in the study as a participant-observer [50]. That is, the author took part in the preparation of the co-design sessions by ensuring the participation of FUs and by exploring different ways of integrating conversion factors, facilitating the co-design sessions, debriefing co-design sessions, and developing the prototype from the results of the co-design sessions. In addition, once the co-design phase was completed, she listened to all the recordings of the preparation sessions, co-design sessions, and debriefings; condensed the data; and analyzed the results.

### Recruitment

A secondary data analysis had been planned for and included in the QADA project protocol. As data collection for this study was based on the research team’s work sessions and co-design sessions, no additional recruitment was expected. The researchers adopted a purposive sampling strategy. Community workers were recruited directly. For HSSPs, an email was sent to managers of the participating institutions, who put the team in contact with interested professionals. Caregivers were recruited through community workers and HSSPs. See the paper on the results of the project for more details [51].

### Data Collection

To achieve the targeted goal, the review of conversion factors stretched over several stages of the QADA project:

1. Preparatory meetings for the co-design sessions (including the advisory committee) by the research team (n=24). These meetings provided information regarding the efforts made to ensure optimal mobilization of participants, obtain consensual decision making, and choose the information to be presented to take account of conversion factors. The resulting documents (co-design session planning) and the audio recording of these meetings were used as raw data for the analysis.
  Table 1 presents the number of preparation sessions required for each of the co-design meetings.
2. Co-design sessions (n=8 co-design sessions and *n=*3 working sessions of the advisory committee). In these sessions, information relating to conversion factors was produced to support the effective utilization of the eHealth tool. The sociodemographic data of the participants (provided by them), the resulting documents (artifacts), the audio recordings (of subgroup activities), and the videos of these meetings served as raw data for the analysis. Details of each of the co-design sessions, also presented in a paper on the QADA project [51], are summarized in Table 1.
3. Co-design postsession debriefing meetings (n=11). These meetings helped *us* to quickly identify the perception of the researchers regarding the process and the conversion factors. Note-taking during debriefing and audio recordings also served as raw data for *the * analysis. These meetings took place immediately after each co-design session.

**Figure 2:**
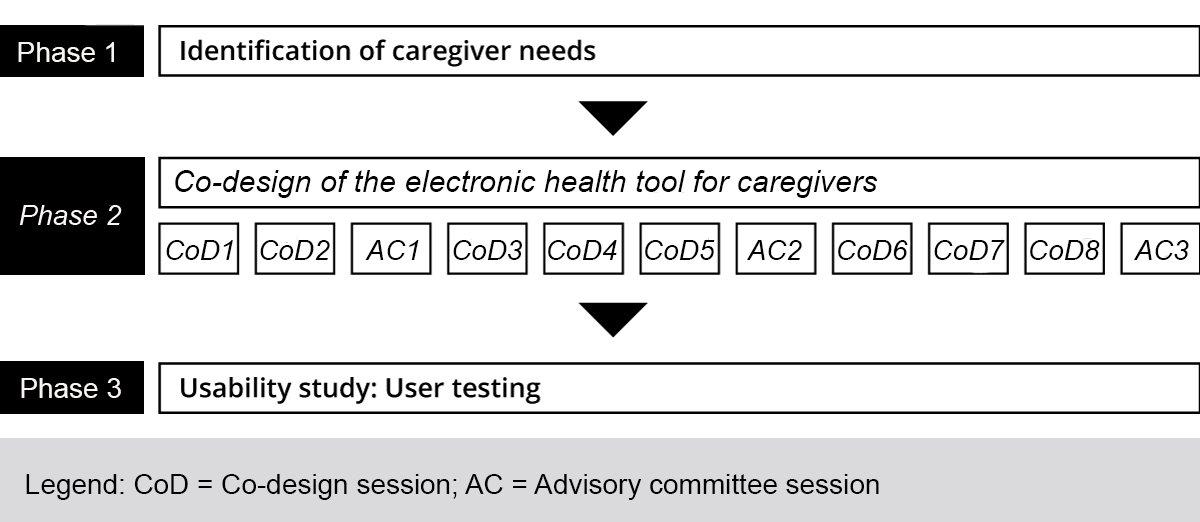
Design Phase of the overall project.

Figure 2 illustrates where this paper fits into the overall QADA project process (in italics).

**Table 1:** Content covered in the co-design sessions and the number of preparation sessions required.

Working sessions	Number of preparation sessions that were required^a^ (n=24)	Content of the co-design or advisory committee session
CoD1^b^	2	Identification of the needs that the tool must address
CoD2	1	Idem
AC1^c^	1	Final choice of needs and recommendations for further co-design
CoD3	1	Exploring existing functionalities that meet needs and identifying gaps between what exists and previously identified needs
CoD4	2	Brainstorming on the functionalities to address the needs that former attempts failed to meet
CoD5	3	Choice of functionalities to be integrated into the tool and development of the site architecture
AC2	1	Choice of functionalities that failed to draw a consensus
CoD6	3	Functionalities and content development
CoD7	5	Functionalities and content development
CoD8	3	Functionalities, content development, and pretest
AC3	2	Exploration of the prototype, choice of more or less realistic functionalities, and discussion on the content

^a^The number of preparation sessions was not defined in advance, but rather defined on an as-needed basis depending on the evolution of the prototype and the complexity of the analysis of the results.

^b^CoD: co-design sessions.

^c^AC: advisory committee.

### Data Analysis

The analysis plan followed the method proposed by Miles and Huberman in 3 convergent analysis activities: data condensation, data presentation, and conclusions development or verification [52].

The purpose of data condensation is to “sort, distinguish, reject and organize data so that final conclusions can be drawn and verified” [52]. In this study, data condensation resulted in a written summary of each document and audio or video recording concerning the preparation of the co-design sessions, the co-design sessions themselves, and the debriefing. Consequently, an initial analysis was carried out to determine what will be reported in the summary document. This choice was made on the basis of the following question: Does what is said in the recording provide relevant information about one of the conversion factors? If so, then the extract was transcribed into a Word document. The reflections emerging in the process of drafting the summary were written in commentary mode. The documents were then imported into MAXQDA software (VERBI GmbH) [53]. MAXQDA is a qualitative analysis software that allows to analyze written documents as well as audio, photos, and videos. A deductive coding was performed to associate the content of the summary documents with the 7 conversion factors. The same extract can be coded with 2 factors. These conversion factors, although independently presented, have several areas of convergence. They were analyzed separately first to see how they will be operationally integrated into the development of the tool and subsequently for their co-occurrence.

Data presentation is an organized collection of information that also aims to draw conclusions. It is presented in the form of tables, cognitive maps, and matrices [52]. In this study, cognitive maps were used to understand how the conversion factors could be considered in the tool as well as the relationships among them. The data were presented in a tabular form to examine the flow of events, the importance given to the conversion factors according to the moment, and progression of the prototype [52].

The development and verification of conclusions occurs when the researcher makes sense of things [40]. These findings, however, need to be rigorously verified by peer review (intersubjective consensus) or by replicating one result into another set of data (triangulation) [40]. As the latter was difficult to produce in this study, the summary documents, cognitive maps, and tables were presented and discussed with the author’s two thesis supervisors. In addition, the accuracy of the summary documents was verified by the other member of the research team (an anthropologist and research professional) who participated in the working sessions, co-design, and debriefing. Considering the large amount of raw data, she checked the accuracy of 10% (1/10) of the documents, at random. She also checked whether the content of the documents was consistent with her perception of the sessions. This study is the subject of a thesis, and it is, therefore, supervised by a thesis committee comprising 4 university researchers in the fields of education and health.

### Ethical Considerations

This project was approved by the *Comité d'éthique de la recherche des Centres de santé et de services sociaux de la Vieille-Capitale* (Research Ethics Committee of the Health and Social Service Centers of the Old Capital). A monetary compensation of Can $20 (US $14.98) was given to each co-designer through the QADA project. The informed consent of each co-designer was obtained in writing.

### Confidentiality of Data and Anonymity

The data were anonymized from the first level of analysis. All the study materials, including information, consent forms, and recordings, were kept in a locked filing cabinet in a locked room at the research center. The digital data were saved in encrypted files, on a secure server of Laval University, and access to it was protected by the use of a password available only to the members of the research team. Finally, all the materials and data will be kept for 5 years and then destroyed.

## Results

### Co-Designer Characteristics

A total of 78 co-designers participated in co-design sessions or advisory committee sessions. Table 2 presents the characteristics of the people who contributed to this study.

**Table 2:** Description of co-designers.

Sociodemographic characteristics		Caregivers (n=30)	Community workers (n=26)	Health professionals (n=18)	Research team (n=4)
**Sex, n (%)**					
	Women	26 (87)	20 (77)	18 (100)	4 (100)
	Men	4 (13)	(23)	0 (0)	0 (0)
**Age (years),** **mean (SD)**					
	42-88	77.9 (11.0)	N/A^a^	N/A	N/A
	24-66	N/A	44.8 (12.3)	N/A	N/A
	29-53	N/A	N/A	39.6 (7.9)	N/A
	33-45	N/A	N/A	N/A	40.7 (5.4)
**Education level, n**					
	Elementary school	1	0	0	0
	High school	10	1	0	0
	College	4	4	6	0
	Vocational studies	1	0	3	0
	University	12	21	9	4
	None	1	0	0	0
	Not mentioned	1	0	0	0
**Age of the relative (years), mean (SD)**					
	61-96	78.2 (9.9)	N/A	N/A	N/A
**Relationship with the person cared for, n**					
	Children	8	N/A	N/A	N/A
	Sibling	3	N/A	N/A	N/A
	Spouse	17	N/A	N/A	N/A
	Friend	2	N/A	N/A	N/A

^a^N/A: not applicable.

### Overview of the Presence of Conversion Factors in the Co-Design Phase

**Figure 3:**
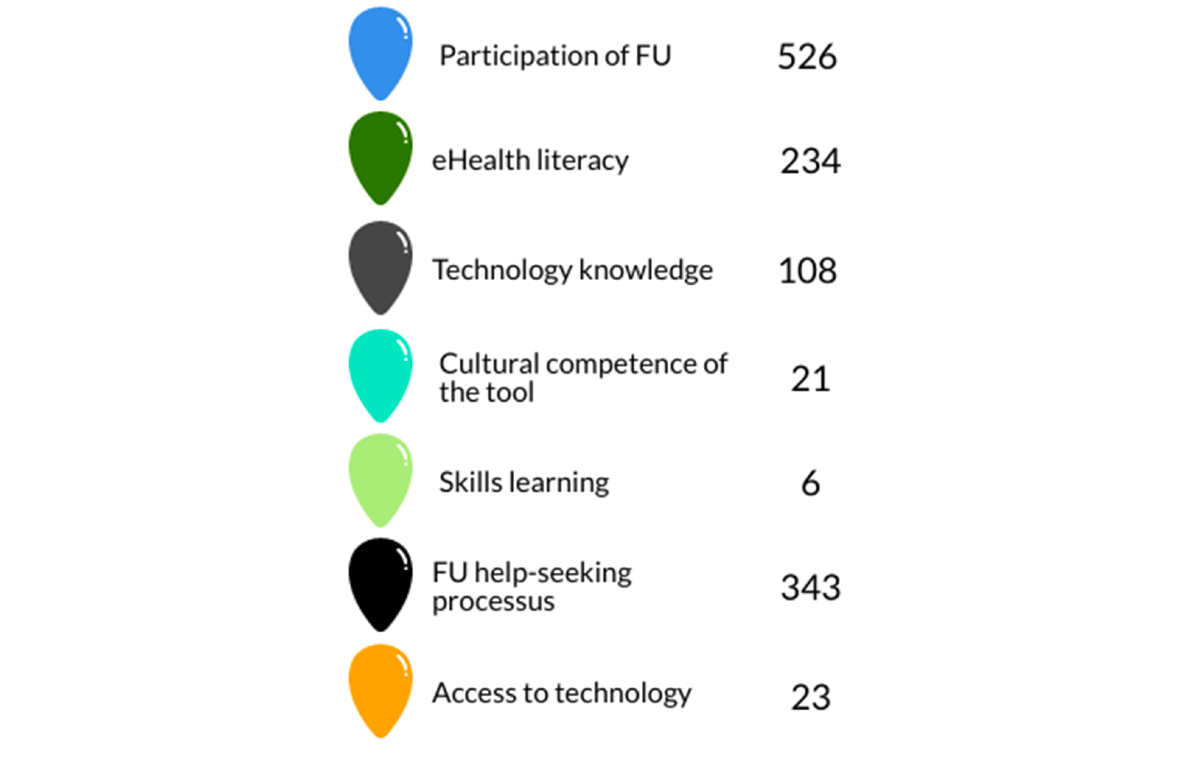
Code list. FU: Future users.

The initial segmentation generated 1257 analytical units. It can be seen that the conversion factors did not have the same occurrence in the development of the tool (Figure 3). Conversion factors were also represented unevenly over time (Multimedia Appendix 1).

The conversion factors, that is, FU participation, eHealth literacy, and the process of seeking help from the FU, were the most discussed in the co-design sessions. Each of these will be the subject of a full paper to reflect the wealth of knowledge resulting from this project. However, we will outline the impact of their integration into the eHealth tool.

### FU Participation

FU participation in the design of the tool was the conversion factor that was present at all stages in a prominent way. This is partly explained by the team’s concern not only by optimizing co-designers’ participation during session preparations but also by respecting the choices they made during sessions. Decision making was coded under this conversion factor. A reflection on this participation and its effect was also the subject of discussion during the debriefing sessions. Co-designers (caregivers, HSSPs, and community workers) were offered the choice to work in large groups or small subgroups and in mixed groups or nonmixed groups (caregivers only, HSSP only, etc) and thus given equal opportunities for expression:

Small groups encourage discussion.

Research team, preparation of CoD1

In small groups, all participants spoke; this was not the case during the plenary session, where one participant did not speak at all.

Research team, debriefing CoD3

Caregiver who did not speak at all during the plenary session but who spoke +++ in small groups with other caregivers.

Research team, debriefing CoD4

We limit the workshops to 60 minutes and we do a plenary session to bring together what was covered in the workshops. We don’t have to, but it’s valuable to see the work of others. One person mentioned that there is no point in a plenary session where no decisions are made. We can hold a plenary session to make group decisions such as choosing the angle to use for the caregiver in the algorithm and let co-designers pick the workshop that most interests them. This way, many people participate in making choices on certain aspects and each person gets to work on what they are most interested in. This is valuable insofar as the groups are fairly balanced. We can add a representativeness criterion (caregiver, community worker, HSSP). We must therefore explain the workshops beforehand.

Research team, preparation of CoD6

#### eHealth Literacy

eHealth literacy was addressed at all stages of the co-design phase, but even more extensively in co-design sessions 6 to 8. This is due to the fact that several content creations were developed at these stages. The creation of content (including word choice and sentence constructions) and also the use of videos, what co-designers see when they look at the prototype, and the concern to have as little text as possible in the tool are manifestations of this conversion factor. There were also interesting discussions on the choice of common words (used by caregivers) versus the new terminology desired for certain diseases (eg, dementia vs neurodegenerative diseases):

The text is very heavy. The first thing I would do is click on the video.

Caregiver CoD7

I am insistent, but I would like “neuro-cognitive disorder” to appear (HSSP). Yes, but you're going to be the only one who knows what it means (caregiver). Caregivers will not know what it is.

Can we put it in parentheses? (HSSP) CoD5

HSSP: what do you mean by category of organization? Team member: it's a community organization, CLSC... Caregiver: you are academics. You have to speak in layman’s terms.

Team member: yes, it's literacy, the other group talks about that. CoD7

Everything on the right has been omitted. We're talking about someone with technological skills. She eliminated what was placed on the right.

Research team, debriefing CoD8

#### Help-Seeking Process

The conversion factor related to help-seeking was significantly addressed in all co-design sessions. This is not surprising considering the nature of the tool that directly addressed this subject, the tool developed in the QADA project to support the process of help-seeking from caregivers. The co-designers provided input on this process at each phase during tool development, especially on the link between the process and the tool. For example, it was discussed that caregivers often sought formal services in a state of extreme emotional exhaustion. The co-designers, therefore, established as a guideline that the tool should provide targeted and complete results (with a brief description and telephone number) in 2 clicks. Consequently, the QADA tool was centered around the search box in the center of the home page:

I did a search on the X website. But sometimes it takes too long. We do it in the evening, we're already tired, it’s too taxing. I think the project must ensure that we can get to the information rapidly and that we can take action rapidly.

Caregiver CoD7

Access (physical, technical, and financial) to eHealth, learning capabilities, technological skills, and cultural context were the conversion factors that were least addressed during the co-design phase of the tool. The results related to these conversion factors are presented in the following sections.

#### Providing Physical, Technical, and Financial Access to eHealth

Access to the tool, although less extensively addressed across sessions compared with other themes, remained a concern throughout the process for both the research team members and the co-designers. The access problems mentioned were based on the assumption that older persons may use technology less, that some people may not have the required skills to use it, or that people in vulnerable situations do not use the internet. Therefore, co-designers feel that the issue of access is linked to the eHealth literacy and technological skills conversion factors. We will return to the relationship between age and technological skills when we discuss this conversion factor:

Some people don't have access to the Internet. The fact that some people are not comfortable with the Internet has nothing to do with age. Of course, making a digital tool excludes people. We can't expect to reach everyone with this. Older adults include three generations.

Researcher AC1

Challenge to create a tool that responds to different people's skills and is interactive.

Caregiver AC1

Team member: You have to think of the isolated and vulnerable person, it has to be simple for them.

Community worker: Well, if you think of the isolated and vulnerable person, well, they don't have the Internet. This doesn’t make sense.

CoD6

The majority of the co-designers mentioned that alternatives to the eHealth tool should be provided for caregivers who do not have access to the internet or technology. Bookmarks with telephone numbers, advertisements, announcements, and posters were proposed to make the tool known to those who do not spontaneously search the internet or to contact someone directly to obtain answers about services for caregivers. Third-party intervention was also identified as a solution to accompany a person who would have an access problem such as a friend, neighbor, or pharmacist:

Caregiver 1: In the other advisory committee, it was said that we need a paper version. Caregiver 2: People can go to the library. Caregiver 1: People who provide home care will not take the time to do this.

AC2

It reminds me of a client who never uses the Web. For this person, it takes a third party, another person who will go online for him.

Community worker CoD1

It takes someone from his circle, a friend, for example, to help him with this.

Caregiver CoD1

You need someone who’s close to him to help with this. The third party will use the tool.

Caregiver CoD1

In addition, co-designers mentioned that mobile technologies may be more accessible today and that the tool must be available for use with an electronic tablet and a smartphone:

It's useful. I did a lot of research on my tablet to find a neurologist and find out how to get my husband evaluated. It's useful because you group everything together. I would like it to be adapted for tablets.

Caregiver CoD8

To date, there is no alternative to the digital tool simply because it is not implemented yet and a transposition into a nondigital format would be hasty. However, it was designed to be used with an electronic tablet and a smartphone:

The alternative will depend on the type of tool developed, so we should wait until we have this information.

Research team CoD3

#### Respecting the Learning Capacities of FUs

Learning capacities were not addressed much for 2 reasons. The first reason is that the tool developed in the QADA project aims to support caregivers in their help-seeking process by helping them find services and by trying to establish contact with organizations. In this sense, eHealth is more of a search help tool than a learning process (even if it requires putting some effort into learning how to browse it). Besides becoming familiar with a new resource, few real-life learning situations are also presented in the tool. The second reason is that tool usability concerns have been further categorized into conversion factors related to digital literacy or technological skills. The motivation and emotions to be considered in the learning process may have been included in the conversion factor related to the help-seeking process given the nature of the tool.

#### Being Sensitive to the FU’s Cultural Context

The conversion factor related to the cultural context of the tool was explicitly expressed in one way. All co-designers mentioned the importance of making the tool available in several languages:

There are Anglophones in Quebec. The site will also be in English, right?

Caregiver CoD5

Will the tool be translated into several languages? Being able to use one’s language is important to identify with the tool.

Community worker CoD6

Several languages: English, Innu, French. It's important.

Community worker CoD1

Among other things, the importance of having a tool in one’s primary language was observed in a co-designing session where co-designers tended to reject sites that were in English because they could not understand them:

As soon as we see that it's in English, bye! We drop them right away.

Community worker CoD3

But in English, I won't read. I would just like some French.

Community worker CoD3

#### Developing an eHealth Tool that Values the Technological Skills of FUs

According to a number of co-designers, age appears to be the main factor explaining poorer technology skills:

The homepage needs to be simplified because caregivers who are seniors themselves are less familiar with technology and they may need to be able to get the information without registering. Many of them do not have an email address.

Community worker CoD5

Have you thought about the fact that older persons are not familiar with technologies? This is very important. I have met some people who are not at all skilled with computers.

Caregiver CoD7

Most caregivers are aged between 40 and 50. I’m my spouse’s caregiver. Even people aged 65 and over are comfortable with the Internet. We must not be ageist. Yes, but there are caregivers who are 80 years old and who do not use the Internet at all. Yes, but these people are supervised, they can go to the library. There are numbers they can call to get help.

Community worker CoD6

The older persons we deal with are not tech-savvy. Forget social media such as Facebook, or email. We have to go to their homes. Phone is okay.

Community worker CoD3

As the participating caregivers of older persons were themselves seniors, there was a concern to make the tool as user-friendly as possible. This appeared prominently in discussions of co-design sessions 3 and 5, where the objective was to identify and choose functionalities for the QADA project tool. Some features have been discarded due to their perceived complexity:

I think most people will go on there and look for information without creating a profile. I'm not attracted to webinars. It's not something I’d do automatically. I am not part of this generation that watches webinars.

Caregiver CoD5

Wanting is not enough. A person in their fifties, who’s used to the Internet, won’t be scared; they’ll be able to answer the questions and they'll get it. For someone who doesn’t use computers, it must be as simple as possible.

Community worker CoD5

HSSP asked a caregiver if she would be comfortable with writing *Caregiver: No, I would call. HSSP: Chatting and BOTS are excluded.*

CoD5

Other features were chosen specifically to accommodate users with poorer technological skills:

I think of my elderly people who end up creating plenty of profiles because they get all mixed up. The tool must have a message that tells the user that their email address is already in the database but the password is incorrect, or that the email is not in the database so they don't create a new profile every time.

Team member preparation CoD7

Make clickable images and buttons obvious by putting them in 3D effect because clickable images are not used by older persons.

Team member preparation CoD7

Some members of the research team wanted to make sure that the tool would be useful both to people who feel at ease with technologies and to people who do not. Till now, they had the impression that participating co-designers had poor skills. Other team members saw this as an advantage because it allowed them to choose features that increased the chances of designing an inclusive tool:

It won't help us to have only caregivers who are not familiar with technologies. But it helps us to simplify as much as possible.

Team members Debriefing CoD4

To date, in our co-designs, it is still caregivers who make little use of the technology and will not use it in the process of help-seeking. This is the reality right now. This is the reality for the spouses, perhaps not the children.

Team member Debriefing CoD4

This concern is all the more relevant given that the profile of caregivers who use the internet is likely to change in the coming years:

[Speaking of BOT] Fifteen years from now, people will be more empowered and may be interested in this feature.

HSSP CA2

I don’t know many caregivers who have iPads and who manage well with them. There are a few, but not many. Of course, caregivers today and caregivers in 20 years' time will not be the same.

Community worker CoD8

### Convergence and Linkage Between Conversion Factors

**Figure 4:**
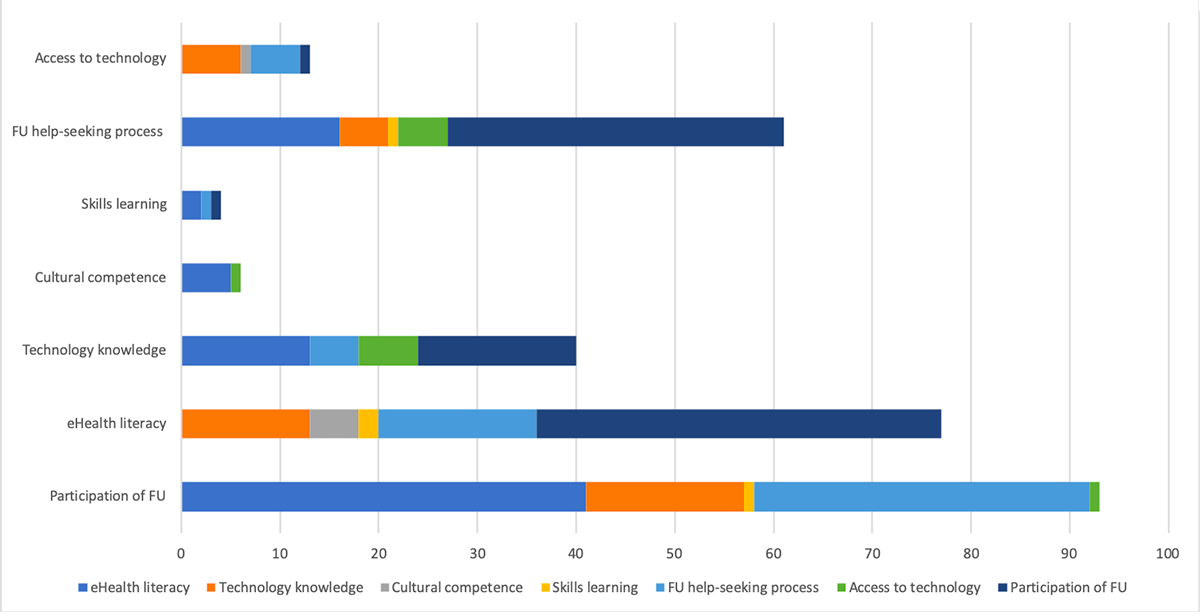
Co-occurence between codes. FU: Future users.

Several verbatim extracts support more than one conversion factor. Figure 4 shows the co-occurrences between the themes.

As discussed earlier, less tech-savvy people may have difficulty using a digital tool. In this sense, several verbatim extracts have been categorized into these 2 conversion factors. The same applies to the process of help-seeking, which may be hampered by limited access to technology and poor technological skills. eHealth literacy is defined as the degree to which individuals have the capacity to obtain, process, and understand basic health information and services needed to make appropriate health decisions. Therefore, technological competence and the help-seeking process should be considered as literacy skills, as reflected in the categorization of verbatim extracts.

It should be noted that there is a co-occurrence of the conversion factor related to FU participation with the other conversion factors. It appears that understanding conversion factors such as eHealth literacy, technological skills, and the help-seeking process requires FU participation in the development of the tool.

## Discussion

### Principal Findings

The objective of this study was to explore how 7 conversion factors can be integrated into the design of an eHealth tool.

The results suggest that conversion factors can be integrated into the development of an eHealth tool at different levels. The technological skills of FUs can be taken into account when choosing the functionalities to be integrated into the tool and the format of the technology used (computer vs tablet). However, these decisions are not easy to make. In this project, the technological skills of caregivers ranged from poor to advanced. Some co-designers did not use technologies because they said they lacked the skills, whereas others indicated that they mastered them very well. One might think that co-designers’ perception was biased by stereotypes related to poor technological competence among older persons. However, statistics show that 19% of adults believe that they have poor skills and they do not use the internet, suggesting that 81% of adults feel that they have moderate to good skills [28]. In addition, individuals’ sense of competence tends to decline with age [28]. As designers, we must, therefore, juggle with heterogeneity in the FU’s skills, while the eHealth tool is intended for the general public or, in this case, for caregivers. This leads to a dilemma faced by the co-designers about the exclusion of features that might have been useful for experienced users but prove to be too complex for neophytes, and also the expected evolution of individuals’ technological skills in the years to come. Indeed, internet use by older persons in Canada has steadily increased between 2007 and 2016 [29]. The presence of more tech-savvy caregivers would have undoubtedly allowed us to discuss this dilemma with them. It would have been appropriate to evaluate the effect of the decisions made on the caregivers’ perception regarding their technological skills during usability tests. Would the complex functionalities that were excluded in favor of more basic ones have allowed caregivers who felt they had poor to moderate technological skills to effectively use the tool? Could we have kept the tool very simple and user-friendly and still included more advanced features accessible to people willing to use them?

The conversion factor related to eHealth access was not addressed by co-designers in terms of access to efficient bandwidth, for example, in rural areas, or in financial terms, but rather from the angle of technological competence and eHealth literacy level. This may reflect the fact that in Quebec, financial or material access to eHealth is not perceived as a major problem. Nevertheless, we must remain cautious and vigilant to ensure that access to eHealth becomes universal because there is concrete evidence that internet access is not globally available (especially in rural areas). Discussions with co-designers led to the exclusion of the use of technology for the so-called less competent individuals rather than adapting the eHealth tool or training them to use it. There seems to be a consensus among caregivers that they are not interested in using technology and that it will be necessary to supplement the tool to support them in their help-seeking process. One of the avenues discussed in this study is to have a third party use the eHealth tool to search for services. This possibility is of particular interest in the context of caregiving because one of the triggers for seeking formal services is also the intervention of a third party (paper in preparation). The third party would act as a mobilizer both to identify the services available through the eHealth tool and to encourage the use of formal services. The inclusiveness of the tool would be further enhanced by its availability to third parties, who can be friends, neighbors, pharmacists, and so on. Instead of referring to access, this conversion factor could be renamed *third-party assistance*.

The conversion factor related to the cultural context was minimally integrated by explicitly addressing the language. The integration of this factor goes further than simply making the tool available in several languages. Can it be argued that this conversion factor was implicitly integrated by involving the FU? Can we assume that the choices made by co-designers were necessarily in accordance with their beliefs, values, and habits? In the case of this experiment, there was little cultural variation among the co-designers as the majority were French Canadian, although this was not what was initially desired. The only variabilities present were related to the particularities of each region, which were integrated by selecting services by sector rather than by region, for example. However, as help-seeking is a process that varies from one cultural community to another, caregivers from an ethnic minority may not feel concerned by the tool [54]. Sen argued that the cultural dimension can only be respected by allowing for debate between users because individual cultural differences persist within the same cultural community [10]. Only the mediation between individual and collective preferences through debate can reconcile differences. The participation of the FU appears to be the way to integrate cultural context into the eHealth tool insofar as co-designers represent cultural diversity.

Similar to the cultural context, knowledge of the caregivers’ help-seeking process was also made possible through the discourses of the co-designers, especially the caregivers themselves. Unlike other design methodologies where the FU is questioned punctually, co-design has allowed us to reflect on the process of help-seeking, which we may not have thought to address in an interview or questionnaire. Co-design allowed the tool to ensure that each of its development stages was consistent and adapted to the caregivers’ help-seeking process.

For eHealth literacy, the team used literature to help developers ensure that their tool would require no more than basic literacy. Elements include language, cognitive overload, and visual exploration by people with low literacy skills, among others, with cross-references to conversion factors such as technological skills and learning abilities. In concrete terms, it is through the content of the site (the choice of words and sentences) that we were able to keep the literacy level requirement to a specific level. However, the simple and refined nature of the tool intended by co-designers, especially in relation to the conversion factor related to technological skills, is also consistent with the principles found in the reference documents related to literacy.

In summary, conversion factors, particularly compliance with the desired eHealth literacy level, the help-seeking process, and cultural context, were integrated into the eHealth tool by the co-designers’ discourse and, more importantly, by the caregivers themselves.

In future research on conversion factors, it does not seem useful to continue to focus on the 7 factors. A range of population-based measures are underway to ensure physical and financial access to technology, and a number of alternatives (eg, free access in shopping malls), although imperfect, are now available. Efforts should be streamlined to pressuring governments to guarantee access to technologies for all citizens in the same way that it ensures access to hydroelectricity, for example. However, it seems essential to continue to look for solutions to the access problems related to technological skills. Nevertheless, this issue can be addressed under the conversion factor related to eHealth literacy. According to some authors, eHealth literacy is composed of 2 types of skills: general skills that include traditional literacy (reading, writing, and numeracy), media literacy (media analysis skills), and information literacy (information seeking and understanding) as well as specific skills that include computer literacy (IT skills), health literacy (health knowledge comprehension), and science literacy (scientific processes and outcomes) [26,55]. Learning abilities, eHealth literacy level, and technological skills of FUs are closely related; it is, therefore, difficult to distinguish the respect of each separate element. From an operational perspective, eHealth literacy assessment could include technological skills as well as, perhaps, learning abilities.

**Figure 5:**
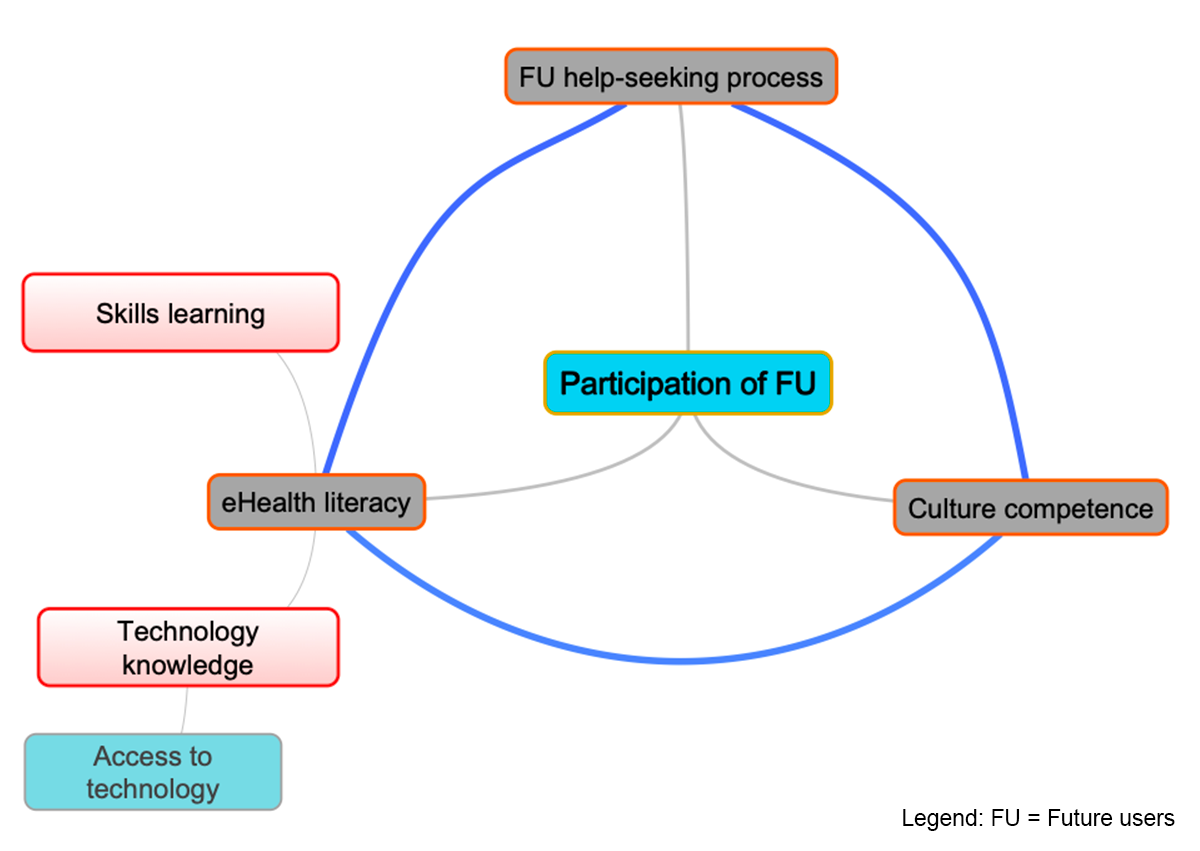
Relationship between conversion factors.

If we had to map the conversion factors to be considered in the development of an eHealth tool to date, here is what it would look like (Figure 5).

The participation of the FU would be the central conversion factor that allows the integration of the other conversion factors. Learning abilities would likely be an integral part of the concept of eHealth literacy. However, the risk associated with this integration would be to leave out the findings generated by the project from the perspective of education and cognitive science about learning in the digital context. The interconnectedness of access to technology, technological skills, and eHealth literacy may raise questions about whether these conversion factors are all necessary or whether they could be functionally grouped under a single concept. However, similar to learning abilities, there would be a risk of losing all the knowledge related to these concepts, each of which could be the subject of future research.

### Scientific Quality of the Study

The rigorous approach of this study is based on the scientific quality criteria identified by Guba and Lincoln [56] for the field of qualitative studies.

#### Credibility and Dependability

To ensure the credibility and dependability of the study, data collection was spread over a period of 1.5 years and it involved a variety of participants (caregivers, community workers, HSSPs, and research team members) having various profiles (gender, age, comfort level with technology, etc). In addition, various data were collected from the recordings of the preparation, co-design, and debriefing sessions. The consistency between data and results is supported by the supervision of 2 researchers (the author's thesis supervisors), one of whom was not involved with the project. The reader was invited to judge the consistency between the verbatim excerpts and the results presented. In addition, the links between the data (synthesis documents) and the coding (deductive and inductive) were also made available to the supervisors. Finally, the synthesis documents were verified by theoretical triangulation and by researchers (KL and MC).

#### Transferability

Transferability was ensured by producing as complete a description as possible of the contexts related to the research process, including the profile of the participants. The reader will, thus, be able to determine the degree of transferability of the results of this research in other contexts.

#### Dependability and Confirmability

The first author (KL) used a reflective approach by highlighting her preconceived ideas, participating in each debriefing meeting, and adding comments to the documents.

### Limitations

This study has some limitations. As already mentioned, cultural diversity could not be represented through co-designers, which limited the possibility of studying the conversion factor in relation to the cultural context. In addition, the caregivers who participated as co-designers were mostly retirees and hence not representative of caregivers among the active population. These caregivers could have influenced the study of conversion factors related to the help-seeking process, technological competence, and eHealth literacy. Similarly, most of the caregivers recruited were already service users. Caregivers at the beginning of the help-seeking process could also have contributed to the study of the process-related conversion factor. Although in line with Quebec statistics, where the majority of caregivers are women (approximately 58%) [57], as is the case for community workers (approximately 80%) [58] and health service providers (approximately 64%) [59], there was no variability regarding gender in our sample. It is possible that greater gender diversity could have influenced the occurrence of conversion factors. Another limitation, and recommendation, for those who wish to develop an eHealth tool with conversion factors is the participation of information technology resources, programmers, and so on as co-designers and team members. This would help them to become familiar with the viewpoint of other co-designers such as caregivers. It would also allow co-designers to better explore the various possibilities offered by functionalities to meet the needs of the caregivers. However, the sharing of decision-making power in such a context will need to be rigorously monitored.

Finally, the evolving nature of the project related to the development of an eHealth tool and the inherent chronology meant that data saturation was not obtained for each of the conversion factors. However, this was anticipated given the exploratory nature of this project.

### Benefits of the Project

This project contributed to the empirical exploration of 7 conversion factors and to the modeling of the relationship among them. Bonvin and Farvaque [13] argued that the link between resources (capital) and capabilities (ie, conversion factors and free choice) is poorly developed by Sen and requires more empirical exploration. Thus, although the strength of the capability approach is to capture social injustice, it provides little evidence as to how to practically address social injustice in communities [60]. This point is also supported by Lorgelly [61], Bonvin [62], and Kleine [63], who pointed out the absence of a modus operandi (planning, implementation, and evaluation). Thus, this project has contributed to operationalizing Sen’s theoretical framework of social justice in a digital context and further developing the concept of conversion factors.

### Conclusions

Conversion factors can be integrated into the development of eHealth tools that are intended to be inclusive and contribute to the reduction of SHIs by integrating the participation of FUs into the design of the tool. However, there is currently no way for the developers of the eHealth tool to rapidly and effectively ascertain whether these conversion factors are well integrated into the development of their tool. The growing development of eHealth around the world, especially in this time of a pandemic, and the governments’ commitment to combating SHIs provide a unique opportunity to reflect on good practices for an inclusive and healthy digital society. To pursue this reflection, it will be important to identify empirical indicators that can measure these integration factors during and after eHealth tool development and guide developers in the designing of inclusive eHealth tools and educational technology that genuinely contribute to reducing SHIs.

## Appendix

Multimedia Appendix 1 Conversion factors over time.

## Abbreviations

FU: future user
HSSP: health and social service professional
QADA: Québec ami des aînés (Age-Friendly Quebec Program).
SHI: social health inequality
