# Supplementary figures and images for: Integration of Conversion Factors for the Development of an Inclusive eHealth Tool With Caregivers of Functionally Dependent Older Persons: Social Justice Design
Source: JMIR Hum Factors. 2020 Aug 26;7(3):e18120. doi: 10.2196/18120 (PMC7481878; doi:10.2196/18120)

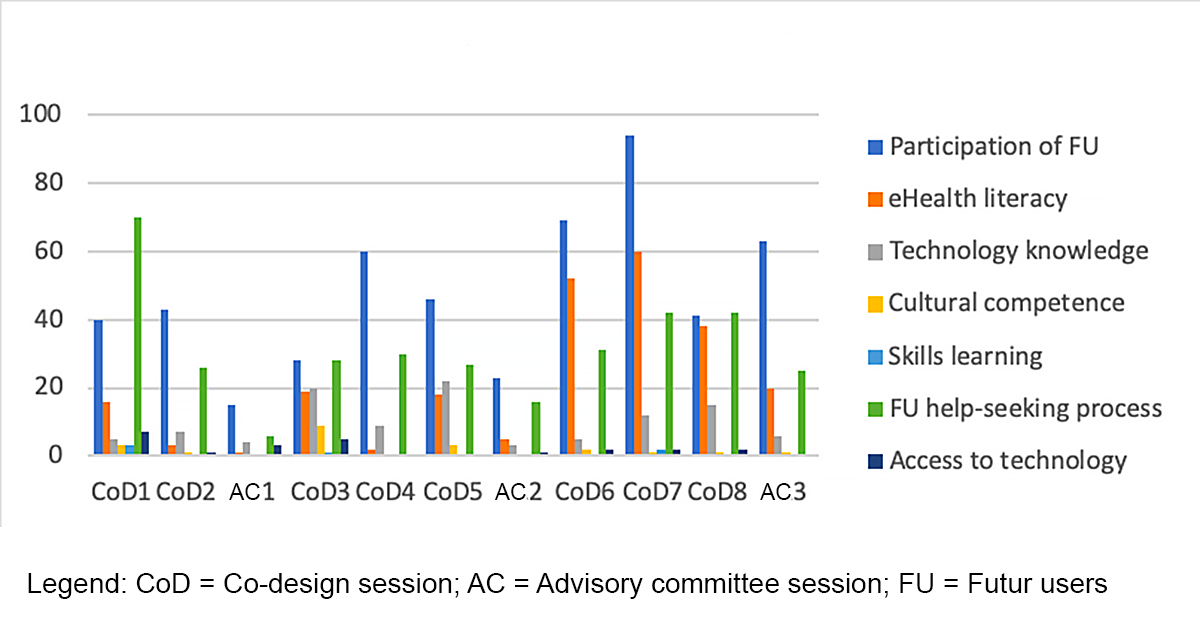

Supplement: Multimedia Appendix 1 [file humanfactors_v7i3e18120_app1.png]
